# Toward Dynamical Modeling of Infants' Looking Times

**DOI:** 10.1002/wcs.70006

**Published:** 2025-05-24

**Authors:** Ralf Engbert, Josephine Funken, Natalie Boll‐Avetisyan

**Affiliations:** ^1^ Psychology University of Potsdam Potsdam Germany; ^2^ Research Focus Cognitive Science University of Potsdam Potsdam Germany; ^3^ Linguistics University of Potsdam Potsdam Germany

**Keywords:** dynamical models, gaze trajectories, infant development, looking times

## Abstract

Analyzing looking times is among the most important behavioral approaches to studying problems such as infant cognition, perception, or language development. However, process‐based approaches to the dynamics of infants' looking times are lacking. Here, we propose a new dynamical framework for modeling infant gaze behavior with full account of the microstructure (i.e., saccades and fixations). Our process‐based model is illustrated by reproducing inter‐individual differences in a developmental study of noun comprehension (Garrison et al. 2020). In our modeling framework, numerical values of model parameters map onto specific cognitive processes (e.g., attention or working memory) involved in gaze control. Because of the general architecture of the mathematical model and our robust procedures in model inference via Bayesian data assimilation, our framework may find applications in other fields of developmental and cognitive sciences.

## Graphical abstract

A dynamical model reproduces gaze trajectories in infants during looking time experiments, explains inter‐individual differences via parameter variations, and generates hypotheses on mechanisms underlying developmental change.

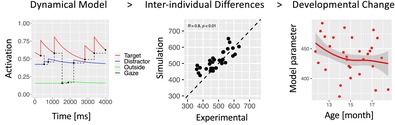

## 1 Introduction

Humans are mainly guided by their vision. Since high‐acuity visual processing is limited to the fovea, a small visual field region centered on the current gaze position, we must direct our eyes to objects of interest, a process termed active vision (Findlay and Gilchrist 2003). Therefore, gaze position informs about ongoing cognition (Rayner 1998). Consequently, looking‐time analyzes are among the most important experimental tools to study infant development in perception, cognition, language, and other research fields (Aslin and Fiser 2005). Looking times are typically operationalized by the relative time the gaze focuses on a target stimulus compared to one or more competing stimuli (e.g., distractors). However, mean looking time represents a global measure (Aslin 2007), selected from a range of other metrics characterizing the *microstructure* of gaze behavior.

Gaze shifts to areas of interest are produced several times per second by saccadic eye movements (Rayner 1998). During eye movements, the brain blocks visual input so that neither the image blur due to the eye's motion nor the gap in visual input is noticeable (Bridgeman et al. 1975). Therefore, almost all information processing is limited to fixations, i.e., epochs where the eyes are relatively motionless. The sequence of saccades and fixations is called a *scan path*. Because there are serial correlations within a scan path, we can extract many conditional variables from eye‐movement recordings. Examples are stimulus‐dependent fixation durations (e.g., mean value of all fixation durations on a target vs. a distractor), the probability to refixate a specific stimulus, or saccadic transition rates (e.g., the probability to move from a distractor to the target stimulus), to name a few. Thus, when looking time is considered the only measure of gaze behavior, we discard many informative properties of the experimental data. Most critically, changes in the microstructure of gaze behavior are generally not noticeable in the average looking time. Therefore, Aslin (Aslin 2007) called for research efforts to make use of the microstructure of gaze behavior in developmental science, which generated a number of research papers on the problem (Helo et al. 2016; Ross‐Sheehy et al. 2022; Pomaranski et al. 2021; Renswoude et al. 2019a). These studies established specific findings on the microstructure of gaze behavior. For example, the center bias known from scene exploration behavior is also present in infant data (Renswoude et al. 2019b). There are also theoretical models on the microstructure of gaze behaviors, for example, the WALD‐EM model (Kucharský et al. 2021) and the CRISP model (Nuthmann et al. 2010) applied to infant gaze data (Urabain et al. 2017). Subsequently, it has been argued that analyzing the microstructure of gaze behavior in infants is difficult because experimental data would be too noisy (Aslin 2012). In this perspective, we implement Aslin's (Aslin 2007) research program based on progress in three research areas: analyzes of the microstructure of looking behavior via high‐resolution eye‐tracking, the development of computational models of gaze behavior, and the availability of Bayesian inference for dynamical models (i.e., data assimilation).

First, without the analysis of looking times, “we would know very little about nearly any aspect of infant development” (Aslin (2007), 48). Mean looking‐times represent a universal measure of processing time allocated to a specific visual object or area of interest. Moreover, looking times can be calculated from various observational methods, including video analysis (Fernald et al. 2008) and eye‐tracking. The situation has changed profoundly with the availability of video‐based eye‐tracking with high temporal resolution (> 100 Hz) that provides the possibility to detect saccades (Wass et al. 2014; Renswoude et al. 2018). With saccade detection, the analysis of scan paths produced by infants in experimental settings generates many variables, all related to the specific aspects of cognitive processing (D'Souza et al. 2020) involved in the coordination of gaze behavior (Aslin 2007). As an example, we can exploit the microstructure of gaze behavior to investigate biologically based models of attention (Barbaro et al. 2011). An in‐depth analysis naturally requires more effort (Fassbender 2022), but it also forms the basis for developing and testing process‐based models (Robertson et al. 2004).

Second, eye movement research generated several computational, process‐based models of gaze control (Reichle et al. 2003) over the last 25 years. In our prior research, we developed models that fall into the class of activation‐based models for eye‐movement control in reading (Engbert et al. 2005) and scene‐viewing tasks (Schwetlick et al. 2020) with structural similarity to the neural dynamic field theory (Amari 1977; Erlhagen and Schöner 2002). In this approach, basic units for saccadic selection, such as words, objects, or areas of interest, are represented by temporally evolving activations so that relative activation levels determine probabilities for saccadic target selection (Engbert et al. 2005). Cognitive processing of an item induces a temporal change of its activation. This change of activation is formulated mathematically as an equation of motion (Engbert 2021; Schöner and Spencer 2016). Process‐based models are defined by an initial state and an equation of motion are dynamical systems (Beer 2000), which produce a trajectory of internal states over time from an initial state.

Third, it is a strength of dynamical cognitive models to generate and explain sequential dependencies. The likelihood function, as a most important concept for statistical inference of dynamical models, has a sequential structure (Schütt et al. 2017) and serves as a basis for Bayesian data assimilation (Engbert et al. 2022; Reich and Cotter 2015). This approach is powerful because the sequential dependencies in the experimental data (the scan path) constrain parameters in the model (Schütt et al. 2017; Engbert et al. 2022; Rabe et al. 2021). With recent progress in the application of Bayesian data assimilation, it is now possible to identify model parameters for individual observers (Engbert et al. 2022). This result is a significant advance in eye movement research, where cognitive models typically aim to explain average behavior against summary statistics pooled across dozens of individuals (Reichle et al. 2003; Engbert et al. 2005).

The progress in the above three areas enables us to implement Aslin's (Aslin 2007) research program on process‐based models of looking behavior that account for the microstructure of infant gaze behavior, inter‐individual differences, and developmental changes. The structure of this article is as follows. We propose a dynamical framework for modeling gaze control that reproduces the microstructure of infant looking behavior (Section 2). The model includes specific mechanisms for saccadic selection, memory decay, and control of fixation duration; we might consider the model as a simplified version of activation‐based theories for reading or scene viewing. Parameter inference (Section 3) demonstrates that our model can explain inter‐individual differences and developmental changes. The methods of Bayesian data assimilation are available in the Appendix A, and all source code is made publicly available via a repository in the Open Science Framework (see link below). Finally, we discuss the implications of our model‐based approach for developmental science and provide conclusions of our work (Section 4).

## 2 A Dynamical Model for Looking Times

Experimental paradigms aim for analyses of looking times typically employ few objects or areas of interest. Here we develop a mathematical model for such paradigms. The most important process for gaze dynamics is saccadic selection (Deubel and Schneider 1996), where saccade targets are represented in a neural priority map (Bisley and Mirpour 2019). Neural firing rates in this map are represented by time‐dependent activations in our computational model developed below. Detailed models of gaze control have been developed (Schwetlick et al. 2020); however, in our model, we do not resolve where the eye fixates precisely *within* an area of interest. Because of this AOI‐based approach, our model is not limited by technical issues (e.g., gaze accuracy, eye‐tracker resolution). We will show that dynamical modeling of the microstructure of gaze behavior contributes new insights under realistic experimental scenarios in infant research.

### 2.1 Activation‐Based Saccadic Selection

As the starting assumption of our model, a finite number *N*_a_ of areas of interest (AOI) is represented by activation values $a_{i}\left(t\right)$ with $i=1,2,\ldots ,N_{a}$ that change continuously over time t. In the context of looking time analyzes, we define the three AOIs ($N_{a}=3$) representing the target (i=1), the distractor (i=2), and everything outside target or distractor AOIs (i=3).

The relative activation value of an AOI quantifies an infant's tendency to prepare a saccadic eye movement toward the AOI. Thus, the set of activations $\left\{a_{i}\left(t\right)\right\}$ determines saccadic selection. Specifically, the AOIs are selected as saccade targets with probabilities computed from relative activations, i.e., the probability $\pi_{i}\left(t\right)$ to select AOI i as the saccade target at time t is given by

(1) $$\pi_{i}\left(t|j\right)=\frac{a_{i}^{\nu }\left(t\right)+\rho \delta_{\mathrm{ij}}}{\sum_{k=1}^{N_{a}}\left(a_{k}^{\nu }\left(t\right)+\rho \delta_{\mathrm{kj}}\right)},$$

where the exponent ν introduces a weighting of activations. For example, there is *winner‐takes‐all* selection for ν→∞. Equation (1) is known as Luce's choice rule (Engbert 2021; Luce 2005) for ν=1. We also introduce an additive refixation bias ρ to reproduce the high prevalence of refixations, where we use Kronecker's delta defined as

(2) $$\delta_{\mathrm{ij}}=\left\{\begin{array}{ll} 1, & \;\text{if}\;i=j\;\\ 0, & \;\text{if}\;i\neq j \end{array}\right..$$

Empirically, all three AOIs will be fixated with non‐zero probability without experimental manipulation and/or instruction. We capture this behavior by task‐independent baseline activations, denoted as $a_{0i}$ (i=1,2,3). In our gaze model, we assume that activations higher than baseline, i.e., $a_{i}\left(t\right)>a_{0i}$, decay to their baseline values $a_{0i}$ (i=1,2,3) according to the set of differential equations

(3) $$\frac{\;d\;a_{i}}{\;d\;t}=\omega \left(a_{0i}-a_{i}\left(t\right)\right).$$

As a consequence, an initial activation $a_{i}\left(t=0\right)=A>a_{0i}$ leads to an exponentially decreasing activation (Schwetlick et al. 2020), i.e.,

(4) $$a_{i}\left(t\right)=A\;e^{-\mathrm{\omega t}}+a_{0i}\left(1-e^{-\mathrm{\omega t}}\right),$$

with decay rate ω. Thus, activation higher than baseline asymptotically reach their baseline values $a_{0i}$ for t→∞.

In the following, we assume that the differences in saccade targeting between target and distractor objects develop over time via learning. As a result, we will have increased activations of target objects, so that saccades to the target object will become more likely over time via by Equation (1). Therefore, we assume that baseline activations are equal for target and distractor, $a_{01}=a_{02}$, for young infants. For each saccade to one of the AOIs i, the associated activation $a_{i}\left(t\right)$ is increased by the factor $\lambda_{i}\geq 1$ at time t, i.e.,

(5) $$a_{i}\left(t\right)\mapsto \lambda_{i}\;a_{i}\left(t\right)\;\text{for}\;i=1,2,3.$$

**FIGURE 1:**
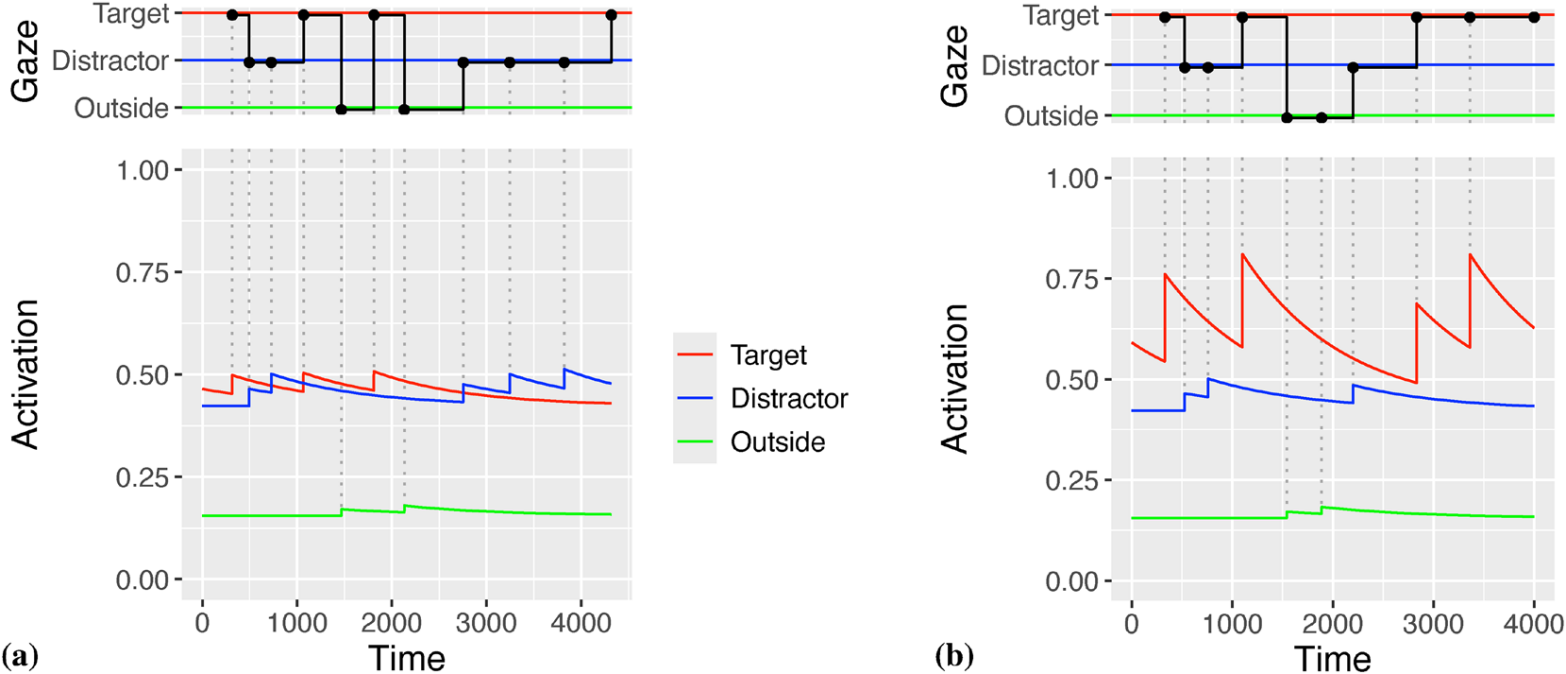
Gaze trajectories simulated by the dynamical model. (a) For younger infants, there is little differentiation between target and distractor which relates to low values of the saccade‐related activation slightly above one ($\lambda_{1,2,3}=1.1$ in the example). (b) For older infants, improved differentiation can be captured by a higher value of the saccade‐related activation for the target object ($\lambda_{1}=1.4$, $\lambda_{2,3}=1.1$ in the plot). *Lower panels*: Activations of target, distractor, and outside regions are indicated by the red, blue, and green lines. *Upper panels*: The gaze trajectory is given by the black line. Saccades are indicated by bullets in the upper panels and by dotted lines in the lower panels.

The λ‐dependent activation of the AOIs introduces a dynamic mechanism of the saccadic selection between the AOIs. There is an additional static component in fixation probabilities represented by differences in the baseline activations $a_{0i}$. Model analyses showed that it is difficult to estimate both the numerical values of $\lambda_{i}$ (dynamical components) and $a_{0i}$ (baselines) simultaneously. Since we are more interested in the dynamical component, we decided to fix the baseline activations at $a_{01}=1.1$ (target), $a_{02}=1$ (distractor), and $a_{03}=0.37$ (outside region). Note that we neglect the saccade duration for simplicity. The resulting behavior is illustrated in Figure 1 by examples for numerical simulations. For infants unable to differentiate between target and distractor, the activations of all AOIs, when fixated, will not increase significantly, i.e., $\lambda_{1,2,3}=1.1$. For those infants who can distinguish between the AOIs, the activation of the target object will increase significantly (e.g., $\lambda_{1}=1.4$ and $\lambda_{2,3}=1.1$ in Figure 1b). This activation increase creates a higher probability for saccades to the target AOI until the activation decays via Equation (4).

In summary for the qualitative behavior of the model, there are activations $\left\{a_{i}\left(t\right)\right\}$ for three AOIs (i=1,2,3). Probabilities for saccadic selection are given by relative activations, Equation (1). The activation $a_{1}\left(t\right)$ of the target AOI is increased by factor $\lambda_{1}$ when the target is fixated. However, activations higher than baseline will decrease exponentially with decay rate ω. For those infants who cannot differentiate between target and distractor (Figure 1a), the activation of the target AOI does not increase substantially over time due to λ≈1. For infants that are able to distinguish between target and the two alternative AOIs, the activation factor is greater than one and the activation of the target AOI increases compared to the activations of the distractor and outside regions (Figure 1b). The activation of the target AOI is transient and undergoes exponential decay. We generally can expect that infants' behaviors also differ with respect to the decay rates ω or saccade targeting exponent δ.

### 2.2 Timing of Fixation Durations

**FIGURE 2:**
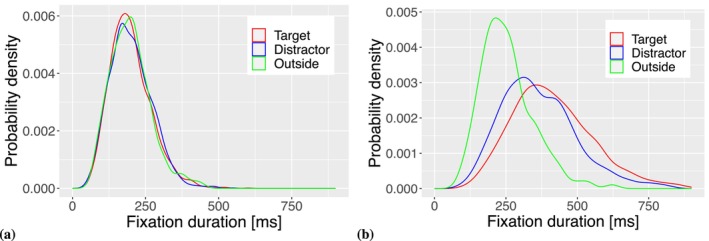
Probability densities of simulated fixation durations. (a) Without inhibition (γ=0), the three fixation duration densities fall on top of each other. (b) If observers are able to control for fixation duration based on activation (γ=0.5), then all three AOIs show specific densities of fixation durations. Data displayed in the plots are generated by model simulations with shape parameter ρ=8 and mean $\mu_{T}=200$ ms for a simulation time of 1000 ms.

It is a well‐established finding that first‐fixation durations only mildly depend on the stimulus features or processing difficulty (Rayner 1998). If more processing time is needed, then the predominant mechanism implemented in the visuomotor system is refixation. Consequently, the gaze duration, i.e., the sum of first‐fixation duration and the durations of all direct refixations, typically depends sensitively on stimulus properties. We demonstrate the presence of this effect in Garrison et al.'s (Garrison et al. 2020) data in Appendix C. Therefore, we start with the assumption of random timing of fixation durations. We assume that fixation durations are realizations of a gamma‐distributed random variable, i.e.,

(6) $$P\left(T\right)=\frac{r^{\sigma }}{\Gamma \left(\sigma \right)}T^{\sigma -1}e^{-\mathrm{rT}}\;\left(T\geq 0\right)$$

with T≥0, shape parameter σ>0 and rate parameter r>0. The generated mean fixation duration is given by $\mu_{T}=\sigma /r$. In the gamma distribution, the shape parameter determines the coefficient of variation $C_{V}$, i.e., the proportion of standard deviation from the mean. It is important to note that the gamma distribution can be replaced by other distributions. Practically, in the R‐language (Ref.), the function for sampling is given by

(7) $$T_{j}∼\text{rgamma}\left(\text{shape},\text{rate}\right)$$

for fixation j, with the two free parameters shape and rate. As mentioned in the beginning of this section, there is mild effect of stimulus saliency on mean fixation duration. In our model, saliencies are given by the set of activations $\left\{a_{i}\left(t\right)\right\}$ for the three AOIs. The activation can also be used to introduce a mechanism of control of fixation durations. For higher activations, we expect longer mean fixation durations. Thus, we replace the mean parameter by a product of the mean and an activation‐dependent factor,

(8) $$\mu_{T}\mapsto \mu_{T}\cdot \left(1+\gamma \;a_{i}\left(t\right)\right),$$

where parameter γ≥0. When parameter γ=0 there is no dependence of fixation duration on AOI, while for γ>0 we observe a separation between the three AOIs (Figure 2). It is important to note that the difference between the densities for the outside region and the distractor AOI is caused by γ due to differences in baseline activations, while the difference between distractor and target AOI is caused by fixation‐dependent activation increase of the target AOI when λ>1.

For an overview of all model parameters see Table A1. In the next section, we will apply the model to experimental data.

## 3 Applying the Model to Experimental Data

Following Aslin's (Aslin 2007) proposal to develop a process‐based research strategy to investigate looking times, we proposed a dynamical model in the previous section. Next, we will illustrate the application of our model using gaze data from an experiment in noun comprehension (Garrison et al. 2020).

### 3.1 The Study on Noun Comprehension by Garrison et al. (2020)

Using a looking time paradigm, Garrison et al. (Garrison et al. 2020) set out to investigate noun comprehension in 12‐ to 18‐month‐old infants.^1^ In the experimental procedure, infants were seated on a caregiver's lap, facing a computer screen. Via headphones, the caregiver heard a test sentence, which they repeated after an auditory signal. An image pair of target and distractor objects were then presented to the infant for 5 s starting with the beginning of pronunciation of the target word. The infant's gaze behavior was recorded via eye‐tracking. Gaze position was classified into three different areas of interest (AOI), the target, the distractor, or the outside region (i.e., all locations not inside target of distractor AOI).

Among the central findings, Garrison et al. (Garrison et al. 2020) reproduced the finding that infants improve their comprehension of nouns in the age range between 12 and 18 months (Bergelson 2020). Following the research strategy in looking time paradigms, the main dependent measure is the proportion of looking time spent on the target compared to the distractor. Figure 2 is a reproduced plot computed from the original data^2^ in comparison to our model simulation discussed below.

### 3.2 Modeling Framework

Our theoretical model is fully implemented on a computer for the quantitative prediction of fixation sequences via numerical simulation (as illustrated in Figure 1). The output of such a *generative model* can be analyzed by exactly the same statistical tools as the experimental data, i.e., the fixation sequences are qualitatively the same as the recorded gaze behavior in the study by Garrison et al. (Garrison et al. 2020). In the following, we apply a fully Bayesian framework for model parameter inference (Engbert 2021; Schad et al. 2021) on individual data sets.

Before parameter inference, we derived and analyzed the model's likelihood function (Section B.1). The likelihood function quantifies the probability of the experimental data under the assumption that our model has generated the data for a given set of model parameters. The important point is the dependence of the likelihood on the set of model parameters. Since a correct implementation of the likelihood computation is critical, we investigated the corresponding profile likelihood (Figure 5). The likelihood function is also important for Bayesian parameter estimation (Schad et al. 2021; Gelman and Rubin 1992). To verify the numerical implementation of our inference framework, we simulated data by our model with known parameters and tried to recover the numerical values of the model parameters from the fixation sequences (Section B.2). This recovery analysis for the model parameters is a critical test of whether sufficient data is available from the experiment, since we simulated exactly the same amount of data as obtained experimentally from one infant. Having passed these methodological tests, we ran simulations to estimate parameters (via Bayesian posteriors) for each infant (Section B.3). Finally, after parameter inference, we sampled numerical values of the model parameters from the posteriors and simulated data, known as *posterior predictive checks*, which we discuss in the next section.

### 3.3 Inter‐Individual Differences

After Bayesian inference (see Appendix B), a probability density over all model parameters, i.e., the *posterior density*, is available for each infant. Sampling from this density permits the generative model to simulate new data for each individual. Since the simulated data and experimental data were qualitatively similar, we could extract the same statistical measures from simulated and experimental fixation sequences.

The goal of our modeling approach is to provide a process‐based explanation of inter‐individual differences via parameter variation in our dynamical model. In this approach, the interaction of changes in model parameters produces inter‐individual differences (see Section 3.4). Before we analyze the parameter changes over age, we first investigate whether our model reproduced the inter‐individual differences. We computed a number of numerical measures that characterize the microstructure of the gaze behavior in experiment and simulation. Figure 4 reports a representative selection of these measures. In Figure 4 a (top rows of panels) different fixation duration measures from simulations are plotted against the corresponding results from experiments. In the left panel, the result for the mean fixation durations are plotted, where each dot represents data from one infant. Ideally, all points would fall on the line of identity (dashed line). The highly significant correlation between simulated and experimental mean values across individuals (r=0.8, p<0.01) indicates that our model captures the inter‐individual differences in the experiment. More specifically, the model also reproduces the mean fixation duration on the target stimulus (center panel, r=0.6, p<0.01) and the mean fixation duration on the distractor (right panel, r=0.66, p<0.01). Therefore, our model reproduces inter‐individual differences in key temporal measures of the microstructure of the infants' looking behaviors via parameter variation.

The spatial microstructure of the gaze trajectories is related to saccadic transition probabilities, e.g., the probability of a saccade from target to target, from target to distractor, from distractor to target etc. A systematic analysis gives a 3×3 matrix of transition probabilities. For each infant, we computed these 9 transition probabilities from experimental and simulated data. Plots of simulated versus experimental transition probabilities are shown in the 9 panels of Figure 4b. Again, each of the dots in the panels represents an individual. The optimal result is achieved when all points are very close to the line of identity. As in Figure 4a, the correlation indicates how well the inter‐individual differences in the experiment were reproduced by the model. An inspection of the 9 panels indicates that 6 of the 9 transition probabilities show highly significant correlations (p<0.01). Thus, we conclude that key features of the inter‐individual differences in the spatial microstructure of the infants' gaze behaviors are reproduced by our model.

**FIGURE 3:**
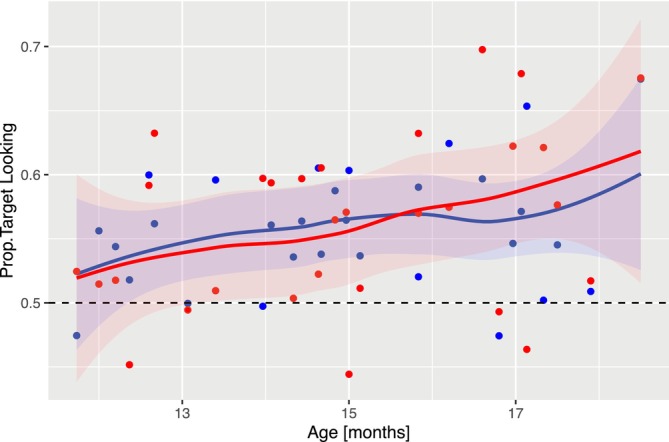
Mean proportion of target looking. Red dots represent individual proportions of target looking calculated from experimental data; blue dots model simulations from posterior predictive simulations (see text). The curves are obtained from locally weighted smoothing.

**FIGURE 4:**
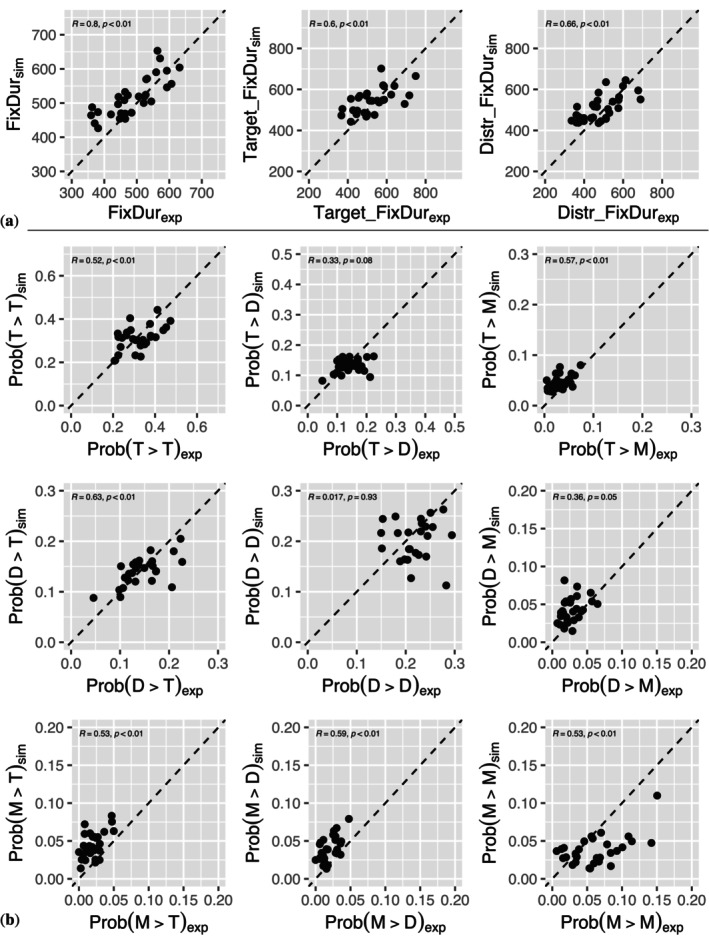
Inter‐individual differences for various measures of fixation duration (vertical axes: Means for simulated data, horizontal axes: Means for experimental data). (a) The top row of panels shows the scatterplots for three different fixation duration measures (for all fixations, target fixations, distractor fixations). (b) The 9 panels below illustrate the results for transition probabilities (T = target, D = distractor, M = missing/outside).

Given our analyzes of the microstructure of simulated gaze trajectories in Figure 4, we will now investigate the proportion of target looking as discussed by Garrison et al. (Garrison et al. 2020). For each individual, we computed the proportion of time the infant was looking at the target stimulus. The proportion is plotted as a function of age in Figure 3 (blue line) and compared to the experimental values (each dot represents an individual). The plot indicates that our model reproduces the experimental effect of an increase in the proportion of target looking with age. Note that the proportion of target looking was not fitted directly. During Bayesian inference of our model, the parameters were optimized to reproduce the microstructure of the gaze trajectories. Based on these individual fits to the data, the model also reproduces the main experimental finding (Garrison et al. 2020).

**FIGURE 5:**
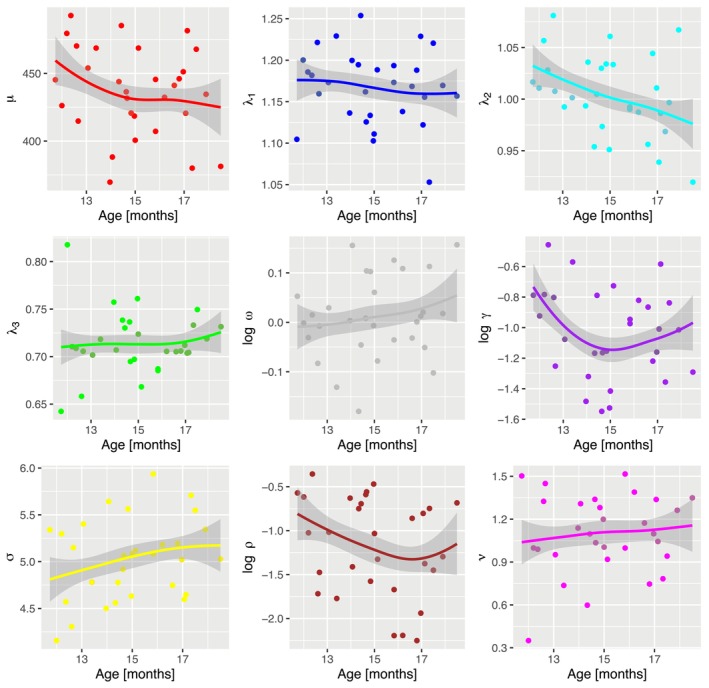
Model parameter changes as a function of age. For each parameter, we calculated a mean model parameter per individual, indicated by the dots in the panels. The lines indicate the trends as a function of age, computed via locally weighted smoothing for illustration.

### 3.4 Developmental Changes

An outstanding feature of process‐based computational models is that parameter changes across age are related to specific component processes in the model (e.g., timing of fixations, build‐up and decay of activation). Figure 5 illustrates the parameter changes across age with locally estimated scatterplot smoothing (LOESS) regression lines added to the panels (see Table A1 for an overview of all model parameters). A glance at the nine panels related to each of the model parameters indicates that there is an age dependence for several of the parameters. The critical effect of the increase in the proportion of target looking (Figure 3) with age is produced by an interaction of several effects. First, dynamic activation parameters $\lambda_{1}$ for the target AOI and $\lambda_{3}$ for the outside AOI are approximately constant. Second, there is a mean decrease in $\lambda_{2}$ for the distractor AOI, while the mean is highest for the target compared to the two other AOIs. Thus, each fixation on the target AOI increases the corresponding activation, which induces a mean increase of the proportion of target looking.

With respect to the specific component processes we observe several additional effects. There is a tendency toward shorter mean fixation durations generated by a decrease of parameter μ with age, which is supported by the previous findings (Renswoude et al. 2019a). This effect coincides with a slight increase in the shape parameter σ. The increase in the decay rate ω with age can be interpreted as an increasing ability to inhibit automatized refixations. Similarly, the decrease in the refixation bias ρ might indicate a shift from refixation habits to active gaze control with increasing age. Change in the remaining parameter might be too premature to be interpreted, and the effects should first be reproduced with independent experimental data. However, some of our current findings translate into plausible effects in the cognitive development of gaze control.

We would like to highlight that the model we are proposing is new, so the results and interpretations discussed above should be considered preliminary; the model needs to be evaluated against several other datasets. The purpose of the current work is to showcase the potential value of process‐based models in comparison to statistical models, and the purpose of Figure 5 is mainly to illustrate how we can learn about process‐based assumptions when a dynamical model is successfully identified (Engbert et al. 2022). Across individuals, we can generate new hypotheses on the relative importance of the model parameters and their underlying processes for explaining developmental changes in behavior. Of course, it is essential to test how the model parameters change over time and whether these changes are plausible against cognitive developmental theories.

## 4 Discussion

We propose and discuss a new dynamical model for infant looking behavior that can account for the microstructure of gaze trajectories (Aslin 2007). The model assumes that activation variables represent different areas of interest (AOIs). First, there is a baseline difference in activations to capture systematic differences between AOIs. Second, there is a dynamical component via saccadic eye movements. Generating a saccade to an AOI transiently increases the activation, which decays afterwards and indicates the strength of working memory representation. We analyze the qualitative properties of the model and parameter identification via Bayesian inference (see Appendix B).

Next, we illustrate the modeling approach using data from Garrison et al.'s (Garrison et al. 2020) study on the cognitive development of word recognition. After model parameter estimation, we simulated gaze trajectories for each individual (posterior predictive checks). Simulated and experimental gaze trajectories are qualitatively similar. We extracted the same measures for fixation durations and saccade targeting (i.e., transition probabilities) from the simulated and experimental data. A comparison of the measure for simulated and experimental data showed that our model captures critical inter‐individual differences in the infants' gaze behaviors in both temporal and spatial aspects of the behavior. Next, we showed that the model simulations also reproduce the experimental finding that the proportion of target looking increases with age (Garrison et al. 2020). Thus, our model successfully couples temporal and spatial control of gaze, including inter‐individual differences for simulating infant gaze behavior; this result is crucial since spatial and temporal pathways for control of saccades are partially independent (Findlay and Walker 1999), but coordination of both pathways is needed to generate purposeful behavior (Kucharský et al. 2021; Engbert et al. 2005).

How does our modeling approach contribute to the analysis of developmental change? After model parameter inference, we are able to analyze the changes of the model parameters with age. While the model was fitted to reproduce individual data, there are also systematic changes of model parameters with age. Since our dynamical model represents a process‐based approach, model parameter changes are linked to changes in component processes. According to our preliminary findings, the looking time effect is based on an interaction of attention control, working memory decay, and improved self‐control.

Finally, developmental theories are inherently dynamical systems (Schöner and Spencer 2016; Beer 2000). For example, Cole et al. (Cole et al. 2019) provide a dynamical systems framework for developmental changes in self‐regulation. While Cole et al. directly link variables extracted from behavior to latent variables of self‐regulation, we propose linking developmental dynamics and self‐regulation via domain‐specific, process‐based models to behavior. In our approach, parameter variations during development represent changes in core cognitive processes, such as attention, working memory, or executive control. In contrast, behavioral indices do not necessarily represent specific underlying processes. Therefore, how model parameters change with age in dynamical models might help us better understand developmental dynamics in perspective.

## Author Contributions

**Ralf Engbert:** conceptualization (equal), funding acquisition (equal), methodology (equal), software (equal), supervision (equal), visualization (equal), writing – original draft (equal). **Josephine Funken:** data curation (equal), methodology (equal), software (equal), writing – review and editing (equal). **Natalie Boll‐Avetisyan:** conceptualization (equal), funding acquisition (equal), methodology (equal), supervision (equal), writing – review and editing (equal).

## Conflicts of Interest

The authors declare no conflicts of interest.

## Related WIREs Articles

Computational perspectives on cognitive development

## Figures and Tables

**FIGURE B1:**
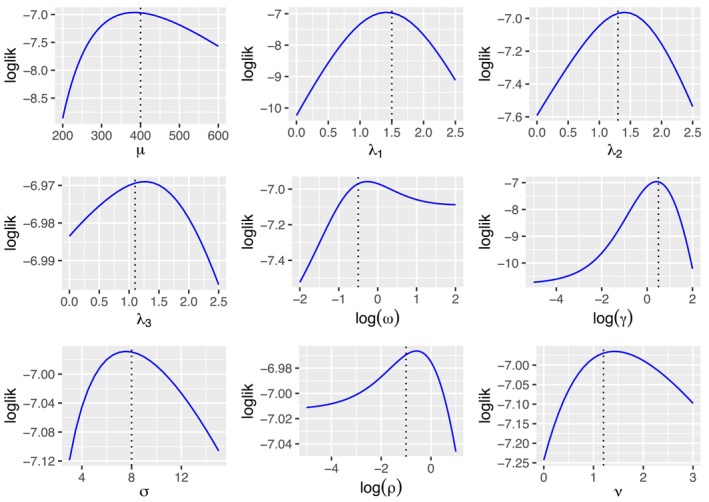
Profile likelihoods for all model parameters of the dynamical model.

**FIGURE B2:**
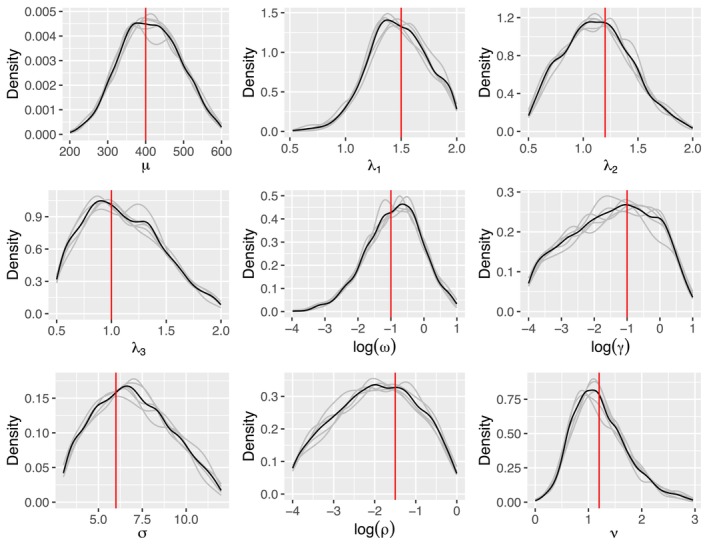
Marginal posteriors and true parameter value obtained from Bayesian recovery analysis.

**FIGURE B3:**
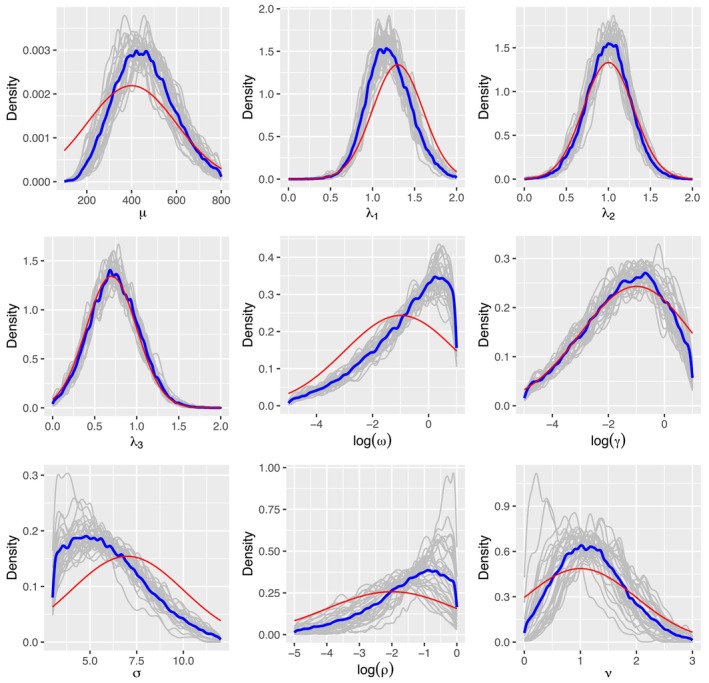
Marginal posterior densities for all estimated model parameters. The red lines represent the priors, the gray lines indicate the posteriors for all individual data sets. The mean marginal posteriors are given by the blue lines.

**FIGURE C1:**
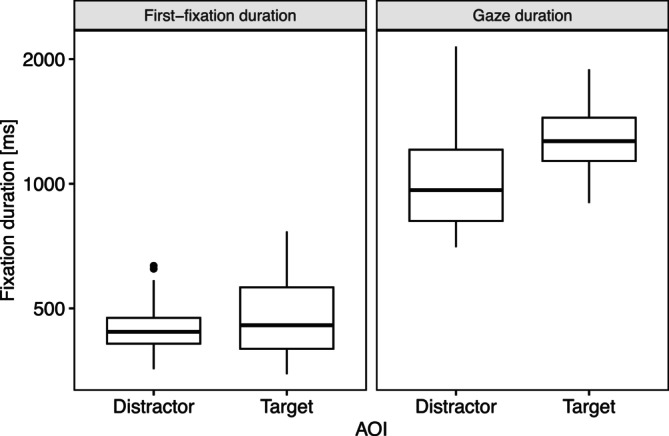
Boxplots for mean first‐fixation durations and mean gaze durations compared for distractors and target stimuli in Garrison et al.'s (2020) data.

## Data Availability

The source code of the model and the reported analyzes are made available via OpenScienceFramework (OSF) at address https://osf.io/bdwau/.

## Endnotes

^1^
It should be noted that the original purpose of Garrison et al.'s (Garrison et al. 2020) study was to investigate the role of familiarity in noun comprehension. The effect of familiarity turned out to be small and the authors conclude that generalization develops early. For details on this aspect of the data, please consult the original publication (Garrison et al. 2020).

^2^
Data were provided by Garrison et al.'s (Garrison et al. 2020) via OpenScienceFramework at address https://osf.io/pb2g6/.

## Appendix A Overview of the Model Parameters

**TABLE A1:** Overview of the model parameters.

Symbol	Description	Reference
$\mu_{T}$	Saccade timer: mean value	Equation (8)
σ	Saccade timer: shape parameter	Equation (6)
$\lambda_{1}$	Dynamic activation: target (AOI_1_)	Equation (5)
$\lambda_{2}$	Dynamic activation: distractor (AOI_2_)	Equation (5)
$\lambda_{3}$	Dynamic activation: missing/outside (AOI_3_)	Equation (5)
ω	Memory decay	Equation (3)
γ	Coupling constant for saccade timer	Equation (8)
ρ	Refixation bias	Equation (1)
ν	Target selection exponent	Equation (1)

## Appendix B Bayesian Data Assimilation

### Sequential Computations of the Model's Likelihood Function

For statistical inference, the likelihood function is a fundamental concept (Myung 2003). Given a set of experimental data, the likelihood $L_{M}\left(\theta |\text{data}\right)$ of a model M with parameters θ is given by the conditional probability $P_{M}$ for the observation of the data, i.e.,

(B1)
$$L_{M}\left(\theta |\;\text{data}\;\right)=P_{M}\left(\;\text{data}\;|\theta \right).$$

For parameter estimation, we are interested in likelihood as a function of the model parameters. In maximum‐likelihood estimation (MLE), we are seeking the set of model parameters θ, which maximizes the likelihood, i.e.,

(B2)
$${\hat{\theta }}_{\mathrm{MLE}}=\arg\max_{\theta \in \Theta }L_{M}\left(\theta |\;\text{data}\;\right).$$

For gaze behavior, the experimental data are sequences of fixations. It is clear from the construction of our model that there will be sequential dependencies in the fixation sequence. Therefore, the likelihood of our model must be computed from the time‐ordered sequence of fixations (Schütt et al. 2017)—a procedure called data assimilation (Reich and Cotter 2015; Asch et al. 2016).

For a fixation sequence ${ℱ}_{S}=\left\{f_{1},f_{2},f_{3},\ldots ,f_{S}\right\}$, we compute the likelihood function sequentially, i.e.,

(B3)
$$L_{M}\left(\theta |\text{data}\right)=L_{M}\left(\theta |f_{1},f_{2},\ldots ,f_{n}\right)=P_{M}\left(f_{1}\right)\cdot \prod_{i=2}^{n}P_{M}\left(f_{i}|f_{1},\ldots ,f_{i-1};\theta \right),$$

where $P_{M}\left(f_{1}\right)$ is the probability of the first fixation t=0. The conditional probabilities $P_{M}\left(f_{i}|f_{1}\ldots f_{i-1},\theta \right)$ are computed by enforcing the model to generate the sequence of fixations ${ℱ}_{i-1}=\left\{f_{1},\ldots ,f_{i-1}\right\}$ to obtain the probability for the $i^{\mathrm{th}}$ fixation $f_{i}$.

We start with an analysis of the likelihood as a function of a single model parameter, as shown in Figure B1 (right column, blue lines). As can be seen from the panels of the right column, the maximum log‐likelihood is found at the true parameter values (dashed black lines). This analysis might be looked upon as a first check of the correct implementation of the computations underlying the likelihood function. The harder problem is the simultaneous estimation of the maximum likelihood, Equation (B2).

In the case of our model of gaze behavior, the likelihood function can be decomposed into a spatial and a temporal component. Each fixation is composed of a fixation location $x_{i}$ (the fixated AOI) and a fixation duration $y_{i}$ (in [ms]), i.e., $f_{i}=\left(x_{i},y_{i}\right)$. Since the fixation duration depends on the selected fixation location, the conditional probability $P_{M}$ in Equation (B3) can be written as

(B4)
$$P_{M}\left(f_{i}|{ℱ}_{i-1};\theta \right)=P_{M}^{\text{temp}}\left(y_{i}|{ℱ}_{i-1},x_{i};\theta \right)\cdot P_{M}^{\text{spat}}\left(x_{i}|{ℱ}_{i-1};\theta \right),$$

with the consequence that log‐likelihood is the sum of spatial and temporal log‐likelihood components (Engbert and Rabe 2024), i.e.,

(B5)
$$l_{M}\left(\theta |{ℱ}_{S}\right)=l_{M}^{\text{temp}}\left(\theta |{ℱ}_{S}\right)+l_{M}^{\text{spat}}\left(\theta |{ℱ}_{S}\right).$$

We increase the spatial log‐likelihood by a factor of 10 for numerical purposes. The spatial likelihood $P_{M}^{\text{spat}}$ for an upcoming fixation position $x_{i}$ is obtained from evaluation of the target selection probability, Equation (1), and, therefore, we can write

(B6)
$$l_{M}^{\text{spat}}\left(\theta |F_{i-1}\right)=\log P_{M}^{\text{spat}}\left(x_{i}|f_{1},f_{2},f_{3},\ldots ,f_{i-1}\right)=\log\;\pi_{x_{i}}\left(t|x_{i-1}\right).$$

Given a fixation location $x_{i}$, the temporal likelihood can be computed by evaluating the theoretical density function of the gamma distribution, Equation (6).

### Parameter Recovery Analysis

In the following, we report some results from Bayesian parameter estimation (Gelman and Rubin 1992) of our model using MCMC simulations. The Bayesian posterior on the model parameter is typically obtained via stochastic simulation methods (Gilks et al. 1995; Robert and Casella 2010). We applied the DREAM  algorithm (Laloy and Vrugt 2012) using 5 chains each for 30,000 iterations.^3^ We simulated 30 trials with total simulation time of 4000 ms, which is representative for the amount of data obtained from a single infant in Garrison et al. (2020) experimental data. Figure B2 visualizes the marginal posterior densities for all 9 model parameters, where the true values are indicated by the red vertical lines. As can be seen from visual inspection, all of the maxima of the marginal posteriors are close to the true parameter values. Thus, our simulations indicate that the parameter can be recovered reliably from simulated data. Moreover, the successful simulation suggest that the amount of data available from the experiment is sufficient to constrain the numerical values of the model parameters.

With a successful parameter recovery we demonstrated the identifiability of our model's parameters. It is still possible that parameter recovery is limited to certain parts of the full model parameter space. One way to secure that a parameter recovery analysis is representative is to run multiple recovery analyzes for all combinations of model parameter value that occur as point estimated for the data under discussion (Rabe et al. 2021).

### Model Parameter Inference

For Bayesian parameter inference on experimental data from Garrison et al.'s (2020) experiment, we carried out MCMC simulations using the DREAM algorithm (Laloy and Vrugt 2012), separately for each individual data set. Figure B3 visualizes the marginal posteriors over model parameters. The priors are indicated by red lines, while the individual marginal posteriors are given by the gray lines. The mean marginal posterior is plotted by the blue lines. The priors were defined as truncated normal densities. Table B2 provides the numerical values of the parameterization of the prior densities. All simulated chains converged according to the Gelman‐Rubin diagnostic, since all $\hat{R}<1.05$. For seven of the nine free model parameter, there is a pronounced difference between prior and posterior densities, which demonstrates that the data constrain most of the model parameters. Also for all parameters we see clear inter‐individual differences in the marginal posteriors.

**TABLE B2:** Overview of prior densities of the model parameters. All priors were defined as truncated normal distributions with mean, standard deviation, minimum, and maximum values. Some of the parameters were log‐transformed (natural logarithm) before parameter estimation.

Symbol	Description	Log	Prior			
			Mean	SD	Min.	Max.
$\mu_{T}$	Saccade timer: mean value	No	400	200	100	800
σ	Saccade timer: shape parameter	No	7	3	3	12
$\lambda_{1}$	Dynamic activation: target (AOI_1_)	No	1.3	0.3	0	2
$\lambda_{2}$	Dynamic activation: distractor (AOI_2_)	No	1.0	0.3	0	2
$\lambda_{3}$	Dynamic activation: missing/outside (AOI_3_)	No	0.7	0.3	0	2
ω	Memory decay	Yes	−1	2	−5	1
γ	Coupling constant for saccade timer	Yes	−1	2	−5	1
ρ	Refixation bias	Yes	−2	2	−5	0
ν	Target selection exponent	No	1	1	0	3

## Appendix C Stimulus Effects on Mean First‐Fixation Duration and Gaze Duration

The effects of stimulus properties on fixation durations are specific to the measure of fixation duration considered. First‐fixation durations typically depend only mildly on stimulus properties, while effects of the stimulus are often strong for gaze durations (Rayner 1998). For the experimental data publihed by Garrison et al.'s (2020), we analyzed the average first‐fixations duration versus average gaze durations (i.e., the sum of first fixation and all direct refixations). Figure C1 indicates that the mean first‐fixation duration does not differ between distractors and target stimuli (P=0.31), but there is a significant difference in mean gaze durations for distractors compared to target stimuli (P<0.001).
